# Identification of mothers with mental health problems is accidental: perceptions of health care providers on availability, access, and support for maternal mental health care for adolescent mothers in Malawi

**DOI:** 10.1186/s12913-024-11469-z

**Published:** 2024-08-26

**Authors:** Chimwemwe Tembo, Linda Portsmouth, Sharyn Burns

**Affiliations:** 1 Saint John of God Hospitaller services, Mzuzu, Malawi; 2 School of Population Health, Faculty of Health Sciences, Curtin University, Perth, Australia

**Keywords:** Mental health services, Access to mental health, Adolescent mothers, Depression, Anxiety, Perinatal, Postnatal

## Abstract

**Background:**

Integration of maternal mental health into primary health care is considered a strategy to improve access to mental health support in low- and middle-income countries (LMICs). Health care workers’ (HCWs) and traditional practitioners’ (traditional healers, herbalists, traditional birth attendants, spiritual healers, prophets, and community health volunteers) perceptions of the availability and management of adolescent mothers’ maternal mental health care were explored in rural Malawi. Recognizing and identifying the barriers associated with access to maternal mental health support is essential to improving the mental health of adolescent mothers.

**Methods:**

A descriptive qualitative design (DQ) was used to explore HCWs’ and traditional practitioners’ perceptions of maternal mental health services for adolescent mothers. In-depth interviews were conducted with HCWs (*n* = 6), and three focus group discussions were conducted with 30 community-based traditional practitioners. Participants were purposefully recruited because they provide formal or informal health services to adolescent mothers during the postnatal period at Mitundu Rural Hospital and its catchment area in Lilongwe District, in Malawi. Interviews were analyzed using reflective thematic analysis and inductive thinking.

**Results:**

Thematic analysis found four themes to describe access to maternal mental health care for adolescent mothers. Participant perceptions were themed around health system challenges and how cultural background and beliefs influence access to mental health care and support. The themes were: (1) Inadequate staff development; (2) Limited resources (medication and infrastructure); (3) Limited policy and guidelines implementation; and (4) Cultural background and belief influence on help-seeking. HCWs suggested improving pre-service and in-service training to improve mental health assessment of mothers, while traditional practitioners wanted to increase their awareness of mental health issues.

**Conclusion:**

Participants emphasized that multifaceted factors influenced access to mental health support. These factors affect the assessment, treatment, and support of adolescent mothers and help-seeking by mothers. Therefore, strengthening the healthcare system and empowering providers with the knowledge and skills to recognize at-risk mothers and provide timely support is essential.

**Supplementary Information:**

The online version contains supplementary material available at 10.1186/s12913-024-11469-z.

## Background

The burden of poor maternal mental health in adolescent mothers is a public health concern [1]. Worldwide, about 10% of pregnant women and 13% of those that have given birth, experience some mental disorder [2]. The prevalence rates of CMDs among perinatal women are two to three times more prevalent in Low to Middle-Income Countries (LMIC) compared to High-Income Countries (HIC) [1, 2]. Studies in LMIC reported a prevalence rate of 19.8% after childbirth [2]. In sub-Saharan Africa (SSA), the prevalence rates of CMDs range from 10 to 39% postnatally [1, 3]. In Malawi, the prevalence of depressive disorders during the perinatal period ranges from 21 − 42% [4, 5]. A recent study in Malawi found a prevalence of postnatal depression of 43.2% among adolescent mothers [6]. Motherhood initiated during adolescence can have its own challenges, hence the urgent need for early identification and treatment of mental health problems. Adolescence brings various challenges and vulnerabilities associated with physical, cognitive, and psychological well-being [7, 8]. For adolescent mothers, this period can be more challenging and may complicate social roles [9, 10]. Considering the broad implications of adolescent motherhood on the well-being of the mother and their child’s development, early identification and effective treatment are essential for preventing the escalation of mental health problems [11]. Untreated maternal mental health is the largest contributor to the disease burden for women of childbearing age [12]. Furthermore, adolescent mothers with untreated postnatal mental health problems are more likely to have low birth weight, poor interactions with their babies, neonatal complications that affect breastfeeding, baby care, parenting and babies may develop malnutrition and frequent hospitalisation [13, 14].

Further, access to healthcare is critical to the performance of healthcare systems globally [15]. However, some challenges exist in LMICs for people with specific health conditions regarding access to comprehensive care [16]. For example, less than half of affected mothers with mental health conditions have access to adequate treatment and healthcare [16]. Mental health care access refers to the opportunity to have mental health care needs fulfilled by having access to appropriate and culturally secure mental health services and/or when communities can use appropriate mental health services in proportion to their needs [15]. The disparity between the number of individuals experiencing mental health issues and the number who receive appropriate treatment is referred to as the treatment gap. In many LMICs, this gap is estimated to be between 75% and 80% [17].

The Government of Malawi is committed to improving health and wellbeing in line with the WHO recommendations and Sustainable Development Goals (SDG) three and five. The Malawian Ministry of Health has developed a mental health policy that aims to provide comprehensive and accessible mental health care services to all citizens of Malawi, including the mental health care needs of special groups [18–20]. However, the implementation of the policy is limited, particularly in managing maternal mental health [18]. The challenges to accessing mental health care in SSA countries and other LMICs derive from inadequate mental health support from the health system [18], economic hardships, a negative societal perception of adolescent motherhood, rural residency, and mothers’ low education levels [12, 18, 21–23]. Additionally, various cultural factors, such as polygamy and traditional practices around gender roles and marriage, impact access to mental health care [24, 25]. Furthermore, adolescent mothers in SSA have lower levels of help-seeking behaviours [24], which has been linked to a perceived lack of confidentiality, negative healthcare worker attitudes, and a lack of services tailored to their unique needs [16]. For example, research with HIV-positive adolescent mothers aged 15–19 years in Malawi exploring barriers and facilitators of prevention of mother-to-child transmission of HIV found adolescent mothers expressed a preference for peer-led, age-appropriate, non-judgmental maternal health support services that link communities and facilities to pragmatically address barriers of stigma, and health system complexity [14]. The Malawian study highlighted the need for tailored services that consider the specific needs of adolescent and young mothers.

Access to healthcare is critical in the management of postnatal depression, and the role of health workers in the prevention of maternal mental health cannot be underestimated [26]. A Malawian study [6] found that adolescent mothers who interacted with a health worker during the postnatal period were less likely to report depression than their peers who did not experience any health worker contact. Interaction with a health worker may provide an opportunity for mental health screening and subsequent referral and support [26]. Perinatal screening is considered an important strategy for the early detection of mental health problems across different healthcare settings and within different economic settings [21, 24, 27]. Furthermore, studies from some LMICs found that perinatal women accepted screening for common mental disorders as part of their primary care [28, 29]. However, there are challenges in implementing screening in some LMICs due to a lack of knowledge among health workers around screening for mental health issues, staff workload, the lack of clinical guidelines for the screening of pregnant and parenting teens, and limited specialized mental health services [18, 26, 30]. Understanding barriers and facilitators to help-seeking is fundamental for developing contextual interventions, enhancing prevention, improving identification, and prompting appropriate treatment [11].

In Malawi, the use of traditional healing is widespread [31], unlike in HIC, where women are more likely to seek help from biomedically trained doctors and midwives [32]. One Malawian study found that the prevalence of people with mental disorders who seek the services of traditional healers before visiting a hospital was 22.7% [33]. Furthermore, another Malawian study found the prevalence of herbal medicine use among all women during pregnancy was estimated at 25.7% in 2018 with use of herbal medicine decreasing among older mothers, with younger women being more likely to use herbal medicine [31]. Another study in Zimbabwe found the use of herbal medicine was more common among women during their first pregnancy or first childbirth [34]. In the context of Malawi, traditional healers provide an alternative or additional approach to mental health care [33]. Therefore, they are key providers in the health care system in Malawi and many other SSA countries [14, 32, 34, 35]. Further, the ethnobotanical research on the traditional use of plants for improving healthcare has been based on knowledge held by traditional practitioners [14, 35]. A study in Mali found that traditional practitioners have broad experience and knowledge about the herbal treatment of pregnant ailments [35]. Understanding mothers’ use of traditional healers can help identify opportunities to address existing access to health services challenges, thereby improving maternal mental health outcomes [31].

The World Health Organization (WHO) recommends the incorporation of mental health prevention, early intervention, and promotion strategies in maternal and child health services to identify at-risk mothers early, therefore reducing the morbidity and mortality associated with poor mental health [36]. Community-based health workers are important in supporting people with their health because they are local, trusted by communities, and integrated within the health system [37]. This study explored the perceptions of healthcare workers (HCWs) and traditional practitioners regarding the access to maternal mental health care by adolescent mothers in rural Malawi.

## Methods

### Setting

The public health care system in Malawi adopts a three-tier health care delivery system based on three levels of health care: primary, secondary, and tertiary [38, 39]. The healthcare system comprises central hospitals, district hospitals, health centers, community hospitals, clinics, and rural hospitals. Each district hospital serves an average population of 600,000 to 900,000 within a geographical distance of 15 to 20 km. District hospitals manage smaller rural hospitals, health centers and health posts [38]. There are twenty-eight administrative districts and twenty-six district hospitals in Malawi (The Malawi Government, 2005). District and rural hospitals operate 24 h, 7 days per week. This study was conducted at Mitundu, located about 40 km east of Lilongwe, the capital city of Malawi. Mitundu is a rural area that has a population of 147,823 [38]. Mitundu Rural Hospital (MRH), the only health facility in the area, was chosen because it serves a relatively higher number of people compared to other facilities within Lilongwe District, with an average of 135 deliveries by adolescent mothers every month. The area has more cases of adolescent marriages compared to urban areas [6]. The hospital also operates thirteen outreach clinics, which are visited monthly by community health workers. MRH provides free outpatient, maternal and child health, family planning and maternity services. The reproductive health services offered at district hospitals include antenatal care, labor and delivery, postnatal care, family planning, cancer screening, and the management of sexually transmitted infections. The facility has forty-six clinical health workers, with eight working in maternal and child health services. Saint John of God Hospitaller Services, Malawi, supports mental health services through conducting monthly outpatient clinics for people with mental illnesses.

### Study design and participants

A qualitative-descriptive design was used to explore the views of HCWs and traditional practitioners’ perceptions of maternal mental health services for adolescent mothers. The HCWs comprised medical practitioners (doctors) and nurses employed in the Maternal and Child Health Department (MCH) at the MRH. Traditional practitioners comprised traditional healers, herbalists, traditional birth attendants, spiritual healers, prophets, and community health volunteers. Community health volunteers are lay community members who liaise with HCWs to assist with health promotion activities in the community.

In-depth interviews were conducted with HCWs to explore their perspectives, experiences and attitudes around their understanding and knowledge of healthcare services and how they are available and accessible. HCWs working in the Maternal and Child Care Department (MCH) were selected as they were in a position to provide a rich insight into appropriate and equitable intervention programs that would meet the needs of postnatal mothers [40]. In-depth interviews allowed participants to share their understanding in a non-threatening environment [40]. Interviews enabled the collection of rich and deep contextual data that could reveal underlying reasons behind delivering maternal mental health services, considering the small number of potential participants [40]. In addition, due to a limited number of HCWs and the need to schedule data collection around work rosters, interviews were deemed the most effective method to collect data from this group.

Traditional practitioners participated in focus group discussions (FGDs), which enabled the discussion of local knowledge and drew on the complex personal experiences, beliefs, perceptions, and attitudes of participants about adolescents’ mental health through moderated interaction [41, 42]. FGDs were employed to gain a greater understanding of traditional practitioners’ role in providing mental health care services [43]. These discussions were used to gather diverse views from multiple participants and allowed for robust discussion [42]. Our study was reported according to the consolidated criteria for reporting qualitative studies COREQ [44] (Supplementary file 3).

### Sampling

A purposive sampling method [45, 46] was used to recruit HCWs who worked in the maternal and child health departments at MRH. The in-depth interviews included six HCWs: two medical doctors, two community nurses, and two registered nurse midwives. All HCWs employed in the Department of Maternal and Child Health at MRH during the data collection period were invited and consented to participate. These HCWs were purposively invited to participate because they are consistently in contact with perinatal mothers during their provision of maternal and child health services [26]. Traditional practitioners were purposively invited to participate in FGDs to achieve variation in gender and the type of services they provide. Thirty traditional practitioners participated in three FGDs and were grouped according to their roles to ensure that participant groups were relatively homogenous. The three FGDs were conducted with traditional healers (six males and four females), community volunteers (eight females and two males), and spiritual healers (three females and one male prophet and six female traditional birth attendants who also use spiritual healing).

### Recruitment of participants and procedures

This study was approved by the Curtin University Human Research Ethics Committee (HRE2021-0223) and the Malawian Ethics Board National Committee on Research Ethics in the Social Sciences and Humanities (P.05/21/575). HCWs were recruited at MRH in their designated work departments. An appointment was scheduled within the hospital at a convenient place and time for each HCW. HCWs were provided with an English version of the information sheet describing the research and procedures and provided consent prior to participating.

Traditional practitioners were recruited through a community health worker who lived within the Mitundu catchment area. An appointment was scheduled to meet potential participants at a convenient community shelter. All participants received information about the study, which was read in Chichewa by the researcher prior to each FGD, and consent was provided prior to participating.

### Data collection

**Fig. 1:**
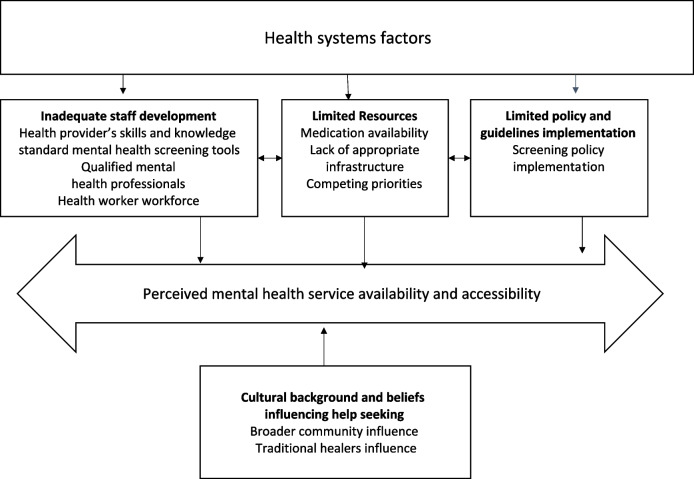
Model describing HCW and traditional healers’ perceptions of availability, access and support for adolescent mothers

Interview guides were used to guide data collection for individual face-to-face semi-structured interviews and FGDs (supplementary files 1 and 2). The interview guides were tested with two HCWs who were not participating in the study to develop the lines of questioning when probing and ensure any ambiguities were excluded and clarified before initiating data collection. Open questions to elicit free responses were used. Questions focused on understanding mental health challenges faced by adolescent mothers during the postnatal period (Fig. 1). The opening questions for HCWs and traditional practitioners explored participants’ understanding of the common mental health problems and disorders that affect adolescent mothers during the postpartum period, with some probing questions to explore further understanding of how common these mental health problems are in the catchment area. Participants were also asked about how they identify and manage adolescent mothers who have mental health problems (supplementary files 1 and 2). The first author conducted all in-depth interviews with HCWs and two of the FGDs. One FGD was facilitated by a research assistant (CN).

Data collection took place between September 2021 and March 2022. Individual interviews were carried out by CT, PK, and CN (Malawian mental health practitioners and researchers fluent in Chichewa and English). The FGD discussions were moderated by CT and CN while PK took notes. The interviews and FGDs were conducted face-to-face, recorded on a digital recorder, and transcribed verbatim. After each interview, member validation was conducted to ensure participant perspectives and experiences were accurately represented. Interviews with HCWs were conducted in English. All FGDs were conducted in Chichewa. Interviews lasted an average of 30 min to an hour, and FGDs lasted 45 min to an hour. Field notes and a reflexive diary that included non-verbal communication and observations were recorded to supplement recorded data.

Prior experiences, assumptions, and beliefs can inevitably impact the data collection [47]. The lead researcher in this study (CT) drew upon her background as someone who experienced adolescent motherhood and is now a mental health nurse in her mid-forties. This experience informed the researcher’s understanding of participants’ perspectives, attitudes, and experiences regarding motherhood. Therefore, the researcher implemented self-reflection and awareness to ensure bracketing. The researcher kept a reflexive diary throughout the data collection process, recording thoughts, emotions, and observations while engaging with participants and gathering data. This served as a resource for analyzing the positionality and potential biases. Additionally, the researcher collaborated with participants to collectively construct shared realities. The construction of reality is a collaborative process between the researcher and the topic under investigation, influenced by the subjective experiences of everyone involved [48]. However, the researcher minimized biased approaches during data collection by holding a neutral stance and not sharing her experiences and views while prompting and guiding the discussions. The aforementioned statements pertain to epistemological presuppositions regarding acquiring knowledge about reality [47].

### Analysis

De-identified transcripts were organised using NVivo version 10 [49] and analysed using thematic analysis [50, 51]. The study employed Braun and Clarke [51] six-phases of reflexive thematic analysis: (1) familiarization with the data; (2) generating initial codes; (3) generating themes; (4) reviewing the themes; (5) final review and defining of themes; and (6) writing the results. During this process, the data was familiarized by reading and re-reading the transcripts and interview notes and noting ideas. Then, using open coding, transcripts were initially coded by (CT) and (MZ) to generate initial codes using an inductive and data-driven approach. CT and MZ coded separately during this process and then discussed the coding. Themes were generated according to the patterns of codes identified, which were grouped and allocated to a theme. The themes were discussed and confirmed by authors SB, CT, and LP to ensure the themes aligned with the coded extracts. Ongoing analysis enabled the refinement of the specifics of each theme and the generation of clear definitions and names of themes and subthemes [50, 52, 53]. The final step was the write-up of the results. Constant comparison by the research team achieved confirmability and dependability. The supervisory team (SB and LP) contributed to the data interpretation and ongoing reviews of the results [54]. The involvement of a team helped remove potential bias and strengthen the results [44].

## Results

Healthcare providers (HCPs) views regarding the availability, accessibility, and management of maternal mental health of adolescent mothers were explored. Participants (*n* = 36) included six HCWs who participated in in-depth individual interviews and 30 traditional practitioners, including 10 traditional healers, 10 community volunteers, six traditional birth attendants and four spiritual healers who participated in the three FGDs (9 males and 21 females). Eighteen participants were Christians, four were Muslims, and eight belonged to traditional religions. Ten participants had never been to school.

### Overview of themes and conceptual model

Four main themes emerged to help understand the perceptions of healthcare workers (HCWs) and traditional practitioners regarding the access and availability of mental health services for adolescent mothers in rural Malawi. The themes of *inadequate staff development*, *limited resources*,* limited policy*, *and guidelines implementation* pertain to health systems. The theme C*ultural background and beliefs influencing seeking help* describes cultural norms.

#### Inadequate staff development

The theme of *inadequate staff development* has four subthemes: *health providers’ skills and knowledge*,* standard mental health screening tools*,* qualified mental health professionals*, and *health worker workforce.*

#### Health provider’s skills and knowledge

All healthcare providers acknowledged encountering many adolescent mothers during their daily activities. HCWs reported focusing on physical examinations, nutrition assessments, nutrition counselling sessions and other health education sessions for pregnant mothers in clinics and at the community level. However, they did not address mental health. However, HCWs perceived themselves to be strategically positioned to provide initial mental health assessments and support to mothers. Some HCWs acknowledged their hesitancy to include mental health assessments as they felt they lacked the skills to identify symptoms of mental health problems and to provide appropriate mental health support. Furthermore, they expressed a need for regular professional development, for example:

*Some of health workers have the skills but… most of us do not have the skills to conduct a mental health assessment…. An in-service or reorientation would be good. Of course*,* we had a training during our clinical placement but…. psychiatric or mental health is difficult*,* we have forgotten (HCW1)*.

Similarly, traditional practitioners also expressed concerns about their limited awareness about mental health and perceived deficiencies in counselling skills. Community volunteers and traditional birth attendants do not routinely include mental health care. Many community volunteers had received prior training in conducting home visits to pregnant women, offering nutrition education, and performing basic physical assessments, which they believed positioned them well to support mothers in crisis before directing them to clinics. While traditional practitioners discussed their need for basic counselling skills, they also recognized the need for experienced counsellors they could refer adolescent mothers to. Other traditional practitioners, such as traditional birth attendants and herbalists, also felt they would benefit from mental health awareness and basic counselling skills.

*The major reason for us who live and work in the community…. We do not have the capacity or basic skills for counselling or communicating with someone about their mental health. We do not have the knowledge for counseling. We need counsellors…. To be honest we don’t teach about mental health but about nutrition only because we don’t have the required skills or knowledge in the mental health field. (Community volunteer FDG1)*

*If there was an opportunity for us to learn how to manage mental health and teach about mental health*,* (it would) be good …. so that we can help adolescent mothers live without stress. We will be incredibly grateful also because we will add more knowledge to what we already teach mothers*. (*Community volunteer FDG1).*

### Standard mental health screening tools

Most HCWs were willing to screen women for possible common mental health problems. However, they attributed the lack of culturally approved screening instruments as a challenge to screening. HCWs felt it important to have a standard screening tool for common disorders incorporated into their routine assessment guides. They reported that most adolescent mothers with common mental health issues requiring help go unrecognized, especially when they look happy or do not show any visible signs of sadness. One health worker summarized this sentiment, suggesting the identification of mothers with mental health problems is mostly “accidental’ due to the lack of formal screening and assessment.

*Most of the time we prioritize those who come here as a family to seek help because we encourage family involvement or partner involvement. So*,* if someone comes alone*,* we explore further to identify the reason she has come alone. That is when we identify the issues. We can say identification is accidental  (HCW3)*.

### Qualified mental health professionals

Most participants acknowledged the importance of specialized mental health professionals who offer tailored and comprehensive assessments and psychological support that incorporate mothers’ specific psychosocial needs. For example,

S*ome mothers needed counselling services and most of them would need psychosocial counselling and psychosocial counselling (which) would range from 1 week to up to 6 months and (this) needs qualified psychosocial counsellors (HCW 2).*

Participants reported that at the time of the study, the hospital had only one mental health nurse working in the labor ward who was responsible for managing clients with severe mental illnesses. To address the issue of limited staff, some HCWs suggested a need for integration of mental health services into primary health care. It was suggested that this could be achieved by engaging non-mental health professionals to support mothers and implement early intervention. This would enable support to be provided to mothers immediately, which was especially important for young mothers, many of whom travel long distances, often on foot, to get to the clinic.

*Another thing*,* is if we can have enough mental health personnel …for example*,* we only have 1 psychiatric nurse*,* the one who deals with all mental health issues… So*,* it will be ideal…if we have … a psychiatric nurse or anyone who looks into all those mental health conditions in the department like for example …. here at antenatal have one-person*,* general wards should have one and the other departments as well. That would help rather than just referring them to one person… It may happen that the person on that particular day is absent he is engaged with other issues so it means these people (adolescent with mental health issues) will not be assisted. And then telling them (adolescents) you should come another day will look like a burden to them. Looking at the distance they walk from home to here. I think that can be a problem. So…If we can have more mental health experts here (HCW 2)*.

### Health worker workforce

HCWs acknowledged the government of Malawi’s commitment to strengthen human resources for health including accelerating training and recruiting health professionals to support all positions required in the health sector. However, some HCWs also expressed concerns over the limited number of staff employed at the facility. Lack of staff was an issue, with the clinic treating around fifty mothers daily with only two nurses per shift, which tended to result in staff prioritizing physical health issues. Some HCWs and community volunteers suggested that community volunteers and community health workers can assist with screening mothers for mental health issues. Community volunteers concurred this would be feasible if they received appropriate training regarding screening procedures and mental health problems.

*Given the workload at the clinic*,* little time is available to screen for mental disorders and hence they go unrecognized…they should be assisted (but) it is only the psychiatric nurse that decides the kind of medication. So*,* most of us will just look at the condition and we do not help much. We only focus on anemia*,* malaria*,* and pregnancy. Furthermore*,* community workers can assist with screening.  (HCW 5).*

*Only if there was that opportunity for us (community volunteer) to learn how to teach about mental health and support mothers so that they could live without stress. We will be incredibly grateful because we will add more knowledge to what we already teach. ….and assist them properly (Community volunteerFGD1)*.

*Yes*,* we can use community workers or can find volunteers in the community who can identify people with mental issues and record their names and bring them to the hospital or provide mental health support (HCW 3).*

### Limited resources

Resource availability was cited as a facilitator for effectively delivering appropriate mental health services. Besides limited human resources, HCWs expressed concerns about the availability of other key resources to deliver the services. This theme has two subthemes: *medication availability*, lack *of appropriate infrastructure. and competing priorities*.

### Medication availability

HCWs reported that all health services provided at the facility are free of charge. Hence, mothers do not pay for consultations and medication received at MRH. The faith-based organization St. John of God Hospitaller Services also partially supports mental health services with a free monthly mental health mobile clinic that provides medications for those with severe mental illnesses who attend the clinic on their clinic day. However, MRH provides perinatal and other outpatient services daily. Therefore, it was challenging for MRH to support mothers who required treatments on non-clinic days because MRH frequently experienced shortages of essential medications used to treat common mental disorders such as depression. HCWs attributed these shortages to limited government financing for medication and that mental health is not considered a priority by the authorities. Additionally, some HCWs also highlighted that medications safe for pregnant and lactating mothers were often not available, leaving some mothers untreated unless they could afford to pay for medication from the pharmacy.

*Sometimes we have capacity*,* but we do not have resources. For example…. having safer antidepressants*,* we rarely have them …. we have the patients*,* but we are failing to put them on safe anti-depression medications… we could ask the family to buy*,* from pharmacies or private clinics… The situation is worse with mental health. No medication at all and we prioritize other medication (HCW 6)*.

### Lack of appropriate infrastructure

Some health workers expressed concern that the current hospital infrastructure does not allow for privacy, making some interventions difficult to implement. Rooms were difficult to access for private counselling sessions. The available open public spaces did not allow confidential discussions and did not have the capacity for partners to attend and accompany their spouses for labor, delivery, and clinic checkups. The need for privacy for mothers who had lost a baby was also emphasized by one participant who expressed concern that these mothers remained on the ward with other mothers and their babies.

*Our hospital physical environment will not allow them (spouses/partners) to come…. postnatally …. they can’t be assisted well if they come with partners ……at least if the facility had rooms to provide privacy (HCW2).*

*We should have a separate room to deal with or to treat the mothers and give counselling because most of them would need psychosocial counselling and psychosocial counselling that would range from 1 week to up to 6 months and (this) needs privacy. (HCW3).*

*Particularly those that have lost their babies we need… a separate room for them because if we put them together with the mothers that have babies that brings in more mental disturbance…being traumatized (HCW6)*.

### Competing priorities

HCWs also expressed concerns over competing priorities with limited funding from the Malawi government and a lack of other non-government organizations and stakeholders supporting mental health services. Participants described how some health conditions receive special donor funding through specific projects. Some of these projects include funding of medication for communicable diseases such as malaria, sexually transmitted infections, and HIV/AIDS. However, there are currently no similar projects that fund mental health medications. This affects the availability of safe treatment options since government funding is insufficient to procure pregnancy-safe antidepressants. Further, during the COVID-19 pandemic, the situation worsened as funding and interventions shifted. For example, *“We noticed that there is a lack of resources*,* particularly drugs because now the focus is on COVID-19 prevention supplies; this has affected the supply of other medications*,* including medication used during emergencies such as adrenaline” (HCW6”).* Another health worker who was involved in providing mental health services stated:

*We have patients who have chronic diseases such as epilepsy and mental illnesses*,* these have been affected more compared to patients that come for malaria or TB treatment Because malaria and TB have specific donors that supply medication but for epilepsy and mental health*,* we don’t have any medications (HCW3)*.

### Limited policy and guidelines implementation

Policy and guidelines were highlighted as facilitators to improved mental health care for women. Nurses and doctors acknowledged the availability of policies and guidelines regarding antenatal and postnatal care. However, the HCWs discussed the gap between these policies and their implementation. HCWs attributed gaps in implementation to issues such as fragmented care and inadequate financing for mental health services, insufficient workforce, unclear practical guidelines specific to maternal mental health care and a lack of staff orientation to new guidelines.

While the Ministry of Health had recently reviewed the antenatal guidelines and incorporated maternal mental health assessment, not all HCWs in this study were aware of these changes. Those who were aware reported that many staff were unfamiliar with recent guidelines. Some participants suggested the guidelines are not explicit, and HCWs require orientation to familiarize themselves with the changes. Furthermore, participants indicated that current antenatal policy does not clearly stipulate the mental health screening of postnatal mothers. HCWs suggested mental health screening should be mandatory.

*Yes*,* the policies and guidelines might be there…so many people (nurses and clinicians) are not even aware of what is in the policies and to use them (policies)…Even the new anti-natal guidelines if you ask some nurses*,* they just know they are there but still practicing old ways where we only assess for physical problems like anemia*,* gestation age*,* and malaria (HCW1).*

*If you may ask me about motivational interviewing*,* and screening*,* I don’t know what it entails (HCW2).*

*I am personally not familiar with the changes; these were not disseminated to us. Orientation would help and for postnatal women we only assess for physical problems up to six weeks postnatal checkup. Probably screening should be mandatory (HCW 3)*.

### Cultural background and beliefs influence on help seeking

Adolescent mothers’ cultural backgrounds and beliefs impacted access to services. *Broader community influence* and *traditional healer’s influence* were sub-themes of this theme.

### Broader community influence and beliefs

Traditional practitioners had different perceptions of how they described mental health problems. Mental health disorders were perceived to be the result of witchcraft or “someone just being silly” or “stupid”, with some participants in the traditional practitioners FGDs suggesting these issues do not warrant hospital treatment. The following quotes from herbalists and a spiritual healer support these sentiments:

*Mental health conditions are because of stupidity mmm …Some people say it’s stupidity …but sometimes it’s indeed witchcraft (Herbalist FDG3).*

*You don’t think straight when you have mental issues. If such things happen*,* some say it is witchcraft*,* some say it is madness*,* and others say it’s Satanism. People talk a lot about these things (Spiritual healer FDG2).*

*……. aah! I think maybe we don’t know that this depression is a condition that can be treated if they can seek help… Only if we know that this is a disease can people go and seek medical help*,* but the problem is that people don’t know that this is a disease (Herbalist FDGs3).*

Traditional practitioners discussed informal support provided within the community rather than the hospital. The discussion with community volunteers highlighted differing approaches. For example, a community volunteer shared an example of an adolescent mother who attempted suicide, refused to breastfeed and abandoned her baby. The mother was taken to the police as laws were broken, instead of being taken to the hospital for mental health support. Some community volunteers agreed with this, for example: *When someone has dumped a baby because the mother is not thinking well …*,* hmm we take the mother to the police station so that the mother is punished” (Community volunteer FDG1).* However, other participants focused on the safety of the child and the mother in this situation, for example: *“It is because they fear for the life of that child. So*,* to protect their lives (the children)*,* they first go to the police station because if the child dies in their hands*,* they (the mothers) may be in trouble. We do not only want to get help for the baby but also save the life of the mother who dumped it. When you get to the police station*,* they tell you to go to the hospital” (Community volunteer FDG1).*

#### Traditional healers influence

Traditional practitioners discussed providing mental health support within rural communities and the importance of traditional social support systems in the context of accessing mental health care. HCWs and traditional practitioners felt mothers seek help from spiritual healers, traditional healers or the health care system based on their perception of their health issue. Therefore, some community members consider traditional healing services the first point of contact for support.

Traditional practitioners reported that some adolescents with symptoms of mental health problems visit traditional practitioners for ‘*breaking of spells’*, a spell being cast by ancestor’s spirits upon the person for wrongdoing with mental health problems as a form of punishment. One traditional healer commented: *“Some of the mental health disorders come when the adolescent’s parents or ancestors did not follow some rituals*,* and therefore*,* they are spiritually tied like a chain…. and this is like a covenant…… and therefore it runs in the families*,* and these can only be healed by breaking the chain….* another one echoed this sentiment: *“They are spells from their ancestors*,* they can only be healed through exhortation” (Traditional healer FGD3)*. A spiritual healer brought a different dimension to dealing with mental issues. For example, one spiritual healer recounted an incident whereby a girl visited the spiritual healer with issues, and the healer felt the girl’s mental health issues were because she was “thinking too much”: *“I had a certain girl at home who was 7 months pregnant. She was always worried when she came to me*,* she never opened up. I do not have a clinic*,* but I practice spiritual healing. She came to me and said you should test me…so I asked her what I should check on. She said just check me. When I consulted the spirits*,* they told me that the girl has no problems in her life*,* but she thinks a lot because of her wrongdoing so she should stop that. I told her that when she stopped thinking a lot*,* her health would improve (Spiritual healer FGD2).* Similarly, another spiritual healer discussed encouraging mothers to talk, pray and make peace with others to alleviate mental health problems:

*When someone with worries comes to me*,* we encourage each other by talking with them through prayers. Then we advise the person on how to behave where she is staying with her neighbours…If some people were not talking to her*,* she should be the first to open up by starting with greeting them. When they do that*,* they come back here to give a testimony…God has helped in resolving the disputes! And most mothers say that …. I thank God and praise him for what He has done because I never thought I would ever be happy again*,* but your prayers and my prayers have worked. God has answered the prayers (Spiritual healer FGD2)*.

In addition, traditional practitioners across all three groups perceived that sometimes, HCWs’ attitude encourages mothers to opt to seek help from traditional healers and other community-based informal providers for their mental health problems rather than HCWs. Traditional practitioners reported that the reception people receive when they visit the hospital is not always positive. For example, in FGD3 with traditional healers, one traditional healer, a traditional birth attendant, stated “*Health workers are the biggest problem*,* so let us be open here. Instead of welcoming and assisting us based on our feelings*,* you treat us badly… (Traditional birth attendant FGD3).* Another Herbalist stated *sometimes you (health workers) take too long to assist someone…instead of assessing someone to know how they are*,* you are busy chatting or sliding your smartphones (Herbalist FGD3).* Similarly, a spiritual healer in FGD2 stated *when adolescents come to us (spiritual healers)*,* we pray for them*,* sit down with them*,* and hold their hands. However*,* in hospitals*,* the care is left to cleaners*,* who sometimes send them back even without seeing a professional health worke*r (S*piritual healer FGD2)*. Given these experiences some traditional practitioners discussed the need to provide better and more compassionate care than hospitals. For example:

*Even though you (health workers) do not allow women to deliver at traditional caregivers… more women around this hospital prefer to go there because they are treated well. The treatment we get from the traditional caregivers and here at the hospital is very different because of the behaviour of the people who work at the hospitals (Traditional birth attendant FGD2*).

*If you put the nurses and the traditional caregivers here and compare them*,* you will see that we manage to help people deliver babies properly in the villages. We treat people very well but at the hospital they are very cruel, they shout at pregnant women (Traditional birth attendant FGD2*).

Notably, all participants from the FGDs and in-depth interviews discussed the need to collaborate with each other. Traditional practitioners felt that traditional healers and birth attendants should refer clients to the hospital. In addition, all traditional practitioners, including community volunteers, wanted more mental health training to enhance their confidence to identify and refer clients with potential mental health problems to the hospital. Participants discussed that while mental health support can be provided by different groups, such as the church, community volunteers, traditional birth attendants, and other people within the community, it was important that mothers experiencing mental health problems were referred to the hospital. HCWs emphasized that proper sensitization and awareness of traditional practitioners and the community around mental health issues is very important due to traditional practitioners’ limited knowledge and skills about maternal mental health. There was a general agreement that task sharing with collaboration is more important than working in isolation. Participants felt utilizing current community structures (community health workers, community health volunteers, and village health committees) would assist in extending support to more mothers. For example:

*The traditional healers and government health workers should work together so that we should encourage the pregnant girls with such conditions (mental health problems) to go to the hospital. So that when they give birth to their baby they can go back to school*,* and this will ensure a better future for those young people and if we see that the girl needs counselling*,* we should take them to the hospital. We should work together because we are all helping people (Herbalist FGD3)*.

*I hope the best lesson is that we should work together*,* we are all helping people including girls. Sometimes we are ignorant of some things*,* do not neglect us, give us trainings so that we refer clients to each other when we meet somethings that we cannot manage. (Traditional Birth Attendant FGD2)*

## Discussion

This is the first study to explore access to maternal mental health of adolescent mothers in Malawi. Access to mental health care services by adolescent mothers remains a challenge in most LMICs [55, 56], and the reasons are multifaceted. In this Malawian study, HCW’s competence in mental health assessment, lack of a culturally appropriate screening tool, limited resources, and cultural and community influences were the perceived factors that impacted access to maternal mental health. Studies conducted in Uganda, Liberia and Nepal around barriers to accessing mental health support for people with mental health problems reported similar findings [57, 58]. In South Africa, the reasons that contributed to poor access to maternal mental health were attributed to systemic health issues such as structural stigma, for example, not implementing appropriate mental health policies, cultural background of mothers, particularly beliefs about the causes of mental health disorders, and health workers competence [11, 59]. Comprehensive access to mental health care requires a multifaceted and holistic approach, highlighting the need to improve mental health literacy and skills among HCWs and traditional practitioners and to address systemic barriers.

While HCWs in this study acknowledged the importance of screening as an essential intervention for early identification and treatment, screening of mothers for mental health problems was challenging. This finding is similar to studies conducted in Kenya and Ghana where health workers also reported challenges in providing screening services due to a lack of training around the conduct of maternal mental health assessments and the lack of a formal process to screen women for postnatal depression [26, 56, 60]. In Malawi, limited competencies among HCWs may be attributed to inadequacies within the current health workers’ pre-service training curriculum. These findings call for a review of the current Malawian nursing and medical curriculum to incorporate modules focusing on screening for common mental disorders in perinatal women. In addition, in-service training for health workers around the implementation of maternal mental health screening tools is essential to improve health workers’ confidence in the provision of comprehensive screening and management.

The findings of this study highlight that insufficient numbers of mental health professionals resulted in fewer opportunities for ongoing support, and long waiting times for mothers to access mental health support. Some studies suggest task shifting to be a strategy to increase access [26]. This involves psychological therapies being provided by non- trained health workers [61] or where tasks are shifted from professionals to community volunteers with fewer qualifications [62, 63], thereby increasing reach [60, 64]. For example, a literature review focusing on the implementation of task sharing in LMICs, where there are limited health workers, has found improved access to mental health support [65]. In Malawi, community health workers, informal health care providers, and community health volunteers are embedded within the community setting. These existing personnel and the current organizational structure may provide opportunities for task sharing [60]. For example, in Malawi, community links with the primary care facility via a team of health surveillance assistants (HSAs), community health workers (CHWs) and traditional healers is possible. HSAs are community-level cadres who receive six weeks of initial training and supervise community volunteers. HSAs and community volunteers provide health promotion and preventive health care through door-to-door visitations and outreach clinics and are supervised by community nurses and public health officers. Each HSA is responsible for a population of 1500, and each village has a volunteer [66]. In this study, HCWs and traditional practitioners suggested a task-sharing approach to provide effective assistance. However, they felt that orientation and training would be necessary considering the complexity of maternal mental health problems and some of the different belief systems and ways of working between HCWs and traditional practitioners. It was evident in this study that traditional practitioners play an important role in the health care of young mothers in Malawi, with many providing examples of support which could be enhanced by training. In addition, policy makers should consider deploying mental health workers in maternal health departments to provide support to adolescent mothers.

Limited resources in terms of infrastructure and competing priorities were also discussed as barriers to the availability of mental health services. These factors affect the type of support available to adolescent mothers. For example, adequate and appropriate infrastructure provides privacy for therapeutic communication and counselling [55]. Lack of infrastructure creates difficulties for both service providers and the consumers of mental health services to render and access appropriate services [55]. Similarly, participants in this study felt that improving the current infrastructure may create a conducive environment that may support adolescent mothers.

Participants in this study highlighted the importance of mental health policy. They felt policies and guidelines on maternal mental health screening in Malawi were not explicit and not well implemented. A lack of policy implementation may contribute to inequality in resource allocation, leading to the diversion of attention and resources, which may result in poor funding of services tailored for maternal mental health. Some authors attribute this to structural stigma within the health system, which affects funding and resource allocation [24, 65, 67, 68]. Therefore, research on effective and efficient mental health interventions would inform policymakers on evidence-based, cost-effective interventions that are appropriate for perinatal women. For example, adopting task sharing using existing community structures and traditional health workers, including community volunteers, could be trialed. Furthermore, policy reviews and enforcement around the implementation of policy may provide support for funding and hence result in improvements in maternal mental health service delivery [69]. The inclusion of screening guidelines in policies would emphasize the need for mental health screening into the postnatal period and facilitate financing for its implementation.

There is ongoing debate on the integration of spirituality or religiosity into the management of people experiencing mental health problems. However, evidence from programs in HIC has found that integrating religious elements into therapy is beneficial for some patients [70]. In the Sub-Saharan African context, [56, 71] and consistent with the findings of this study, traditional practitioners highlighted the benefits of spirituality. Participants in this study did, however, discuss differing beliefs that impacted care. HCWs discussed their biopsychosocial explanation of mental disorders, while some traditional practitioners discussed their belief that bad spirits or being cursed causes mental disorders. These differing beliefs may cause mistrust between HCWs and traditional practitioners. In our study, spiritual healing was reported to assist adolescent mothers in considering life as sacred and meaningful, and as such, was an essential part of the recovery process, with spiritual healers providing alternative coping methods that are essential for healing. Our study also found adolescent mothers who were experiencing mental health difficulties were more likely to be prayerful and hence receptive to intervention from traditional practitioners [6]. These findings highlight the importance of collaborating and supporting religious leaders and other traditional practitioners.

### Strengths and limitations

This study was conducted in a rural setting in Malawi in one hospital catchment area. While findings are unique to this setting, they may be of interest to other regions of Malawi and other SSA countries. The perceptions of HCWs and traditional practitioners provide a unique contribution to the understanding of access and availability of mental health support for adolescent mothers. Despite being conducted during the COVID-19 pandemic, 30 traditional practitioners were recruited. All six HCWs at the MCH at MRH consented to participate in the study.

## Conclusion and recommendations

Access to mental health care support for adolescent mothers in Malawi appears to be impacted by inadequate staff development, limited resources, limited policy and guidelines implementation, and cultural background and beliefs influences on help-seeking. Enhancing mental health literacy among HCWs and traditional practitioners is important to improve their understanding of mental health issues. Training of health workers should include mandatory screening for common mental disorders for pregnant and postnatal adolescents using standard, locally validated screening tools to ensure that mental health problems are detected early. Furthermore, training in empathetic communication skills to improve communication would be essential.

Health facilities should co-develop and implement coordinated interventions with community stakeholders to strengthen community engagement initiatives between HCWs and traditional practitioners with the aim of improving support for adolescent mothers.

Advocacy is required to encourage the Government of Malawi to increase funding for mental health services. Funding is needed to access medications, enhance infrastructure and support policy implementation. Critically staffing increase, especially for mental health nurses, is essential.

Lastly, future research should evaluate the efficacy and effectiveness of interventions that promote help-seeking, enhance prevention, early detection, and timely treatment, utilizing HCWs and traditional practitioners as referral points. For example, research on providing interventions through task sharing would be beneficial.

### Supplementary Information

Supplementary Material 1.

Supplementary Material 2.

Supplementary Material 3.

## Data Availability

Data are hosted by the Curtin University survey office through the Human Research Ethics Committee. Researchers who meet the criteria for access to confidential data can request it through the Chairperson Ethics Committee, and the data will be made available.
