# Supplementary material for: Identification of mothers with mental health problems is accidental: perceptions of health care providers on availability, access, and support for maternal mental health care for adolescent mothers in Malawi
Source: BMC Health Serv Res. 2024 Aug 26;24:983. doi: 10.1186/s12913-024-11469-z (PMC11346021; doi:10.1186/s12913-024-11469-z)
Supplement: Supplementary file 1 — Supplementary Material 1. [file 12913_2024_11469_MOESM1_ESM.pdf]

APPENDIX 1: HEALTHCARE WORKER INTERVIEW GUIDE

In-depth Interview Guide with Healthcare Workers

Demographic details

Age:

Gender

Professional qualification:

Position:

Department:

Address:

Years of clinical service:

Questions related to perinatal common mental disorders.

1. What are the common mental health problems and disorders that affect adolescent mothers during the postpartum period in the area?

Probes: How common are these problems in the catchment area?

2. How do you identify adolescent mothers who have depression and anxiety problems?
3. How do you manage adolescents with perinatal common mental health problems and disorders?
4. Please indicate the extent to which you view the COVID-19 outbreak as having an impact on the mental health of adolescent mothers.
    a. Having a positive impact
    b. Negative impact
5. What would it be if you were to advise adolescent pregnant or new moms during the COVID-19 outbreak?
6. What do you think are the mental health needs of post-partum adolescent women in this area?
7. How has the COVID-19 outbreak changed how you provide services and support to adolescent mothers?
8. In your own experience of working in this area, do adolescent mothers seek help if they have mental health issues? If yes, where do most adolescent mothers with depression or anxiety seek help?
9. In your own opinion, how effective are these helping facilities?
10. How do you collaborate or relate with these traditional services that are in the community
11. What are the social and cultural practices surrounding childbirth among adolescents in this catchment area?
    a. Probe: What cultural practices do you think are supportive
    b. What practices may negatively influence the mental health of adolescent mothers?

12. What is your opinion about mental health interventions that can best help adolescents with perinatal depression or anxiety in this catchment area?
    a. Probe: Pharmacological
    b. Nonpharmacological
13. What is stopping mothers from seeking help?
14. what might assist mothers in accessing mental health support?
15. What programs or interventions are already in place within the community?
16. Can these be utilised/strengthened/upgraded to include a maternal mental health component?
    a. Assess the feasibility/accessibility/willingness of this group to provide support to mothers in distress.
17. Can such interventions be implemented in the community?
    a. Probes: Who/which cadre would best implement the intervention? why
    b. Specialists, PHC workers, Lay volunteers
18. What would hinder the provision of interventions for adolescents with depression and anxiety at the community level using the existing structures?
19. What would facilitate the implementation of interventions to manage common mental problems at the community level?
20. How can mental health interventions be implemented in Maternal and Child Health Services?
21. How confident are you to provide and supervise mental health services?
22. What guidelines are in place to support adolescent mothers during the perinatal period to identify and manage Common Mental Disorders?
23. Are there any plans to integrate mental health services into maternal care?

End of questions
