# Supplementary material for: Identification of mothers with mental health problems is accidental: perceptions of health care providers on availability, access, and support for maternal mental health care for adolescent mothers in Malawi
Source: BMC Health Serv Res. 2024 Aug 26;24:983. doi: 10.1186/s12913-024-11469-z (PMC11346021; doi:10.1186/s12913-024-11469-z)
Supplement: Supplementary file 2 — Supplementary Material 2. [file 12913_2024_11469_MOESM2_ESM.pdf]

INTERVIEW SCHEDULE WITH TRADITIONAL PRACTITIONERS OR INFORMAL HEALTHCARE PROVIDERS (ENGLISH VERSION)

Type of health service (volunteers, traditional healers & traditional birth attendants, prophets & spiritual healers)

INTERVIEW QUESTIONS

1. What does good mental health mean to you?
2. What is your understanding when we say poor mental health or mental disorders?
3. Have you ever seen postpartum adolescents with mental disorders in your community?
    a. Probe: what are the signs and symptoms that they present with?
4. According to your experience, how do you identify adolescent mothers with mental health problems during the postnatal period?
5. How common are mental health problems among adolescent mothers in this community?
6. What do you think are some of the causes of mental disorders in these adolescents during the perinatal period?
7. What do you think are the mental health needs of post-partum adolescent women in this area?
8. What cultural expectations surround childbirth, especially for adolescent mothers in this area?
    a. Probe: What cultural practices do you think may cause mental health problems among adolescents during the postpartum period
    b. What cultural practices may make adolescent mothers have good mental health?
9. What do people say when they see an adolescent with mental disorders during the perinatal period?
    a. Probe: health workers
    b. Families
    c. community
10. Do you have another term or expression that describes poor mental health problems in this community?
11. What happens to those who have mental health problems?
    a. Do they seek help?
    b. If yes, what help do they usually seek?
    c. What support is available for these women in the community?
        i. Probes: from family members
        ii. From the community
12. Where do adolescent mothers with mental health problems seek help?
13. You are health care providers in the community - how do you assist adolescent mothers?
14. From your experience, what prevents adolescents from seeking help?
    a. Probe: from health professionals
    b. From family members and communities
15. What are the challenges to the provision of mental health support, and what do you think should be done to support adolescent mothers with mental health problems?
16.
17. Please indicate the extent to which you view the COVID-19 outbreak as having an impact on the mental health of adolescent mothers.
